# Recovery from Severe COVID-19 Is an Independent Predictor of Electrocardiographic Abnormal P-Wave Axis

**DOI:** 10.3390/diagnostics14131326

**Published:** 2024-06-22

**Authors:** Mücahid Yılmaz, Çetin Mirzaoğlu

**Affiliations:** Department of Cardiology, University of Health Sciences, Elazığ Fethi Sekin City Hospital, 23280 Elazığ, Turkey; dr.cmirzaoglu@gmail.com

**Keywords:** recovery from severe COVİD-19, abnormal P-wave axis, supraventricular arrhythmia

## Abstract

Aim: Abnormal P-wave axis (aPwa) have been correlated with an increased risk of supraventricular arrhythmias. The aim of this study was to analyze whether infection with COVID-19 may cause a predisposition for supraventricular arrhythmia in the long term, following recovery. Materials and Methods: In this study, a total of 252 subjects with a confirmed history of COVID-19 (recovered COVID-19) and 251 healthy subjects without a history of COVID-19 were included. The recovered COVID-19 group was divided into three subgroups designated as mild, moderate, and severe according to the severity score of their chest CT. The aPwa data were obtained using 12-lead electrocardiography (ECG) and compared between the healthy subjects and the recovered COVID-19 subgroups. Results: This study showed that in the recovered severe COVID-19 subgroup the prevalence of aPwa was significantly increased compared to the controls and the other COVID-19 subgroups. No correlation could be detected in Spearman’s Rho correlation between the existence of aPwa and the number of positive PCR tests for COVID-19 and the time elapsed after infection with COVID-19. The binary logistic regression analysis showed that recovery from severe COVID-19, the severity score of the chest CT in the recovered from COVID-19 subjects, and the existence of hypertension (HT) were all independent predictors of aPwa (hazard ratio: 3.542, 95% confidence interval: 1.398–8.969, *p*: 0.01; hazard ratio: 0.896, 95% confidence interval: 0.840–0.955, *p* < 0.001; hazard ratio: 2.710, 95% confidence interval: 1.079–6.804, *p*: 0.03, respectively). Conclusions: Individuals who have recovered from severe COVID-19 have shown an increased prevalence of aPwa. The existence of aPwa was not associated with the number of positive PCR tests for COVID-19 or the time elapsed after infection with COVID-19. Therefore, recovery from severe COVID-19 is an independent predictor of electrocardiographic abnormal P-wave axis.

## 1. Introduction

The new coronavirus (SARS-CoV-2), which emerged towards the end of 2019, quickly obtained a pandemic status and has had a very significant impact on the global health and economic systems. SARS-CoV-2 infection (also called COVID-19) has the potential to cause both pulmonary and systemic inflammation, causing multiple organ failure in the high-risk population [1].

As the COVID-19 recovery rate in the population increased, the symptoms that started to appear and persisted after COVID-19 began to attract more research attention. Long COVID is defined as a syndrome where a set of symptoms persists for more than 12 weeks following acute COVID-19 [2]. In long COVID, the impairment of many systems, including respiratory, cardiovascular, nervous, gastrointestinal, and musculoskeletal, can be observed. The symptoms observed in long COVID, such as fatigue, shortness of breath, chest pain, palpitation, cognitive disorders, attention disorders, arthralgia, myalgia, headache, and hair loss, have been mentioned in some recently published studies [2,3,4,5].

Myocardial damage may occur in patients who were previously infected with COVID-19 and the rates of in-hospital mortality have been high. Direct viral toxicity, inflammation, increased demand, microvascular dysfunction, and plaque rupture caused by SARS-CoV-2 in the myocardium are different mechanisms that are thought to cause cardiac damage and arrhythmia [6,7].

The net direction of the sum of atrial depolarization vectors indicates the involvement of the P-wave axis. The structural and/or electrical remodeling, inflammatory or scar tissue that may occur in the left atrium causes P-wave axis deviation (abnormal P-wave axis). Recently, the abnormal P-wave axis (aPwa) has been used as a marker of supraventricular arrhythmias [8,9].

The question arises as to whether SARS-CoV-2 can cause persistent electrocardiographic (ECG) P-wave axis deviation. In this study, we investigated whether there is a permanent P-wave axis deviation in the ECGs of individuals who were infected with COVID-19 and recovered, and further examined the possibility of supraventricular arrhythmia occurring in these individuals.

## 2. Materials and Methods

### 2.1. Study Population

**Figure 1:**
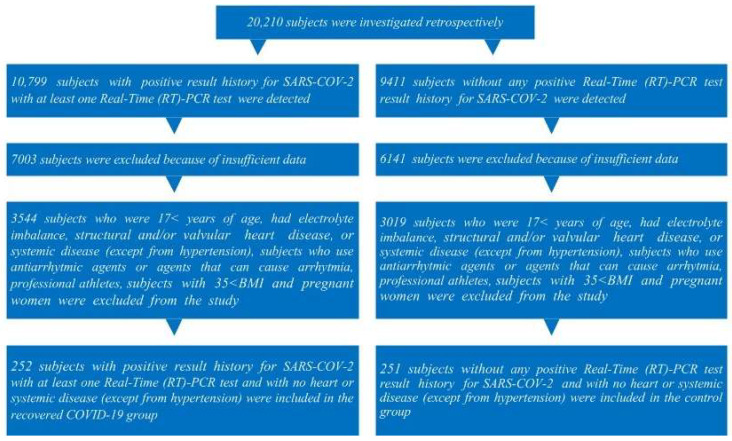
The subject inclusion flowchart diagram.

In this study, the files of a total of 20,210 subjects who were admitted to Elazığ Fethi Sekin City Hospital Cardiology outpatient clinics between 1 October 2021 and 1 May 2023 and underwent a 12-lead ECG record were retrospectively examined. In addition to the patients who had insufficient data in their files, those who were <17 years of age, had atrial fibrillation, electrolyte imbalance, structural heart disease, heart valve disease, or systemic disease (except hypertension), patients who used anti-arrhythmic agents or agents that can cause arrhythmia (such as probucol, terfenadine, amiodarone, erythromycin, clarithromycin, antidepressant agents, antipsychotic agents), professional athletes, subjects with a BMI > 35, and pregnant women were excluded from this study. A total of 252 subjects with positive results for SARS-CoV-2 with at least one nasopharyngeal swab real-time (RT)-PCR test (Bio-Rad CFX96 Real-Time PCR detection system; Bio-Rad Laboratories, Inc., Hercules, CA, USA, ABD) were included in the recovered from COVID-19 group. A total of 251 subjects without any positive (RT)-PCR test result history for SARS-CoV-2 within the same period and whose anamnesis, laboratory, and physical examination could not detect any systemic or heart disease (except hypertension) were included in the control group (Figure 1).

The subjects in the recovered from COVID-19 group had been infected at least once and at most three times with SARS-CoV-2. The interval between the 12-lead ECG examination and the last positive RT-PCR test of individuals in the recovered from COVID-19 group was at least 4 weeks and at most 146 weeks.

A semi-quantitative chest computed tomography (CT) severity score for each of the 5 lung lobes (3 lobes on the right and 2 lobes on the left) was utilized in the group who had recovered from COVID-19. The percentage of the involvement of each lobe was visually evaluated. The chest CT severity score was designed to be between 0 and 25 after multiplying the number of lobes by the percentage of each lobe involvement (Table 1) [10].

The group who had recovered from COVID-19 were classified into three subgroups according to their chest CT severity score. In subgroup I (defined as mild; 155 patients), the score of CT severity was graded as mild (<8). In subgroup II (defined as moderate; 56 patients), the score of CT severity was graded as moderate (8–15). Finally, in subgroup III (defined as severe; 42 patients), the score of CT severity was graded as severe (16–25) (Table 1). 

The present study was conducted in accordance with the principles of the Declaration of Helsinki. The ethical approval was obtained from the Ethics Committee of T.C. Fırat University (No: 2021/12-20). Patient consent was waived due to the retrospective nature of this study.

### 2.2. Laboratory Measurements

The ante-cubital vein was used for gaining samples of 6 mL for full biochemistry testing and 5 mL for total blood count (CBC), following 12 h of fasting. The blood samples were collected in vacuum tubes filled with 15% K3 ethylene diamine tetra acetic acid as an anticoagulant (Sarstedt, Essen, Belgium). The CBC parameters were evaluated using a Sysmex XN-1000 hematology analyzer (Sysmex Europe GmbH, Sysmex Corporation, Hamburg, Germany), according to the manufacturer’s instructions. Venous blood samples were obtained following 12 h of fasting to measure the biochemical parameters using a Cobas^®^ 8000 device (Roche Diagnostics International Ltd., Basel, Switzerland).

Subjects who had taken antihypertensive medication and had a measurement of systolic blood pressure ≥ 140 mmHg and/or diastolic blood pressure ≥90 mmHg were defined as hypertensive. Diabetes mellitus was defined as having a fasting blood glucose level >126 mg/dL (6.99 mmol/L) or the current use of a diet or drug to lower blood glucose levels.

### 2.3. PCR Analysis

In our hospital, PCR analysis for the definitive diagnosis of SARSCoV-2 was performed with the CFX96 Real-Time System (BIO-RAD Laboratories, Foster City, CA, USA).

### 2.4. Chest CT

The chest CT of the patients was obtained using a Philips Integris Allura 9 C monoplane device (Philips Medical Systems, Eindhoven, The Netherlands).

### 2.5. Echocardiographic Measurements

The TTE was conducted with a Vivid 5 instrument with a 2.5-MHz transducer (GE Medical Systems, Milwaukee, WI, USA). The American Society of Echocardiography recommendations were adopted [11].

### 2.6. Electrocardiographic Measurements

For the analysis of the ECG parameters, recordings of the patients taken while in the supine position, resting on the 12-lead ECG device (PageWriter TC20 Cardiograph; Philips Healthcare, Amsterdam, The Netherlands), were used. In these recordings, the filter was set to 100 Hz, the alternating current filter to 60 Hz, the paper flow rate to 25 mm/s, and the amplitude to 10 mm/mV.

**Figure 2:**
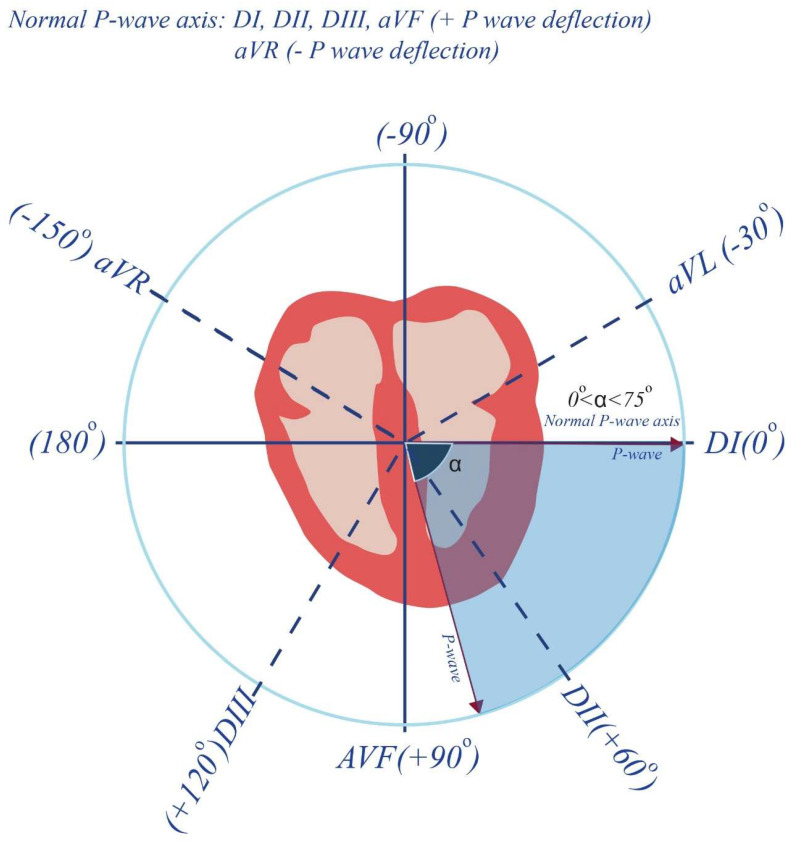
Schematic representation of normal P-wave axis.

Since the ECG device presents the frontal plane P-wave axis, the data were obtained from this position. P-wave axis values between 0° and 75° were accepted as normal and those outside this range were considered abnormal (aPwa) (Figure 2) [12].

### 2.7. Statistical Evaluation

The SPSS 27 package program was used for statistical evaluation. The Kolmogorov–Smirnov test was applied to determine whether or not the continuous variables showed a normal distribution. Since no continuous variable showed a normal distribution, the Mann–Whitney U-test was used to analyze these parameters. The Chi-square test was used for the analysis of categorical variables. The data were presented as median with 25–75th percentiles for the continuous variables, and the categorical variables were expressed as numbers and percentages. Tamhane’s T2 correction was used for the one-way ANOVA test for comparison of the continuous variables. Spearman’s Rho was used to analyze the correlation between the continuous variables. Binary logistic regression analysis was performed to determine which clinical variables independently affected the existence of aPwa. A *p* value < 0.05 was considered significant.

## 3. Results

In this study, 252 patients who had recovered from COVID-19 (the recovered COVID-19 group) and 251 healthy patients with no known systemic disorder except for hypertension (the control group) were included. The recovered COVID-19 group were classified into three subgroups according to the severity score of their Chest CT. The prevalence of aPwa was discovered to be significantly increased in the subgroup that had recovered from severe COVID-19 in comparison to both the controls and the other subgroups (Table 2). On the other hand, no difference in aPwa prevalence was detected between the control group and the mild and moderate subgroups. Finally, although the median age of the cases in the subgroup that had recovered from severe COVID-19 was found to be higher than the other subgroups, no other difference could be detected between the subgroup that recovered from severe COVID-19 and the controls and the other subgroups in terms of smoking, presence of HT, and gender (Table 2).

When the correlation analyses were examined, although a positive correlation was detected between the subgroups that had recovered from COVID-19, the chest CT severity scores for the recovered COVID-19 subjects, and the existence of aPwa, no correlation could be detected between the existence of aPwa, the number of positive PCR tests for COVID-19, and the time elapsed after infection with COVID-19 (r = −0.194, *p:* 0.002, r = −0.245, *p* < 0.0001, respectively) (Table 3).

The results of the binary regression analyses showed that recovering from severe COVID-19, the severity score of the recovered COVID-19 subjects’ chest CT, and the presence of hypertension (HT) were all independent predictors of the existence of aPwa (Table 4 and Table 5).

When Model-1 was examined, it was seen that the binary logistic regression model, established for determining whether the independent variables were effective in predicting the presence of aPwa, was significant (Naegele R^2^ = 0.195; *p* < 0.001). According to this model, recovering from severe COVID-19 represents a 3.542 times risk factor for the presence of aPwa compared to those subjects who have never been infected with the disease or experienced a mild/moderate case of the disease and recovered (hazard ratio: 3.542, 95% confidence interval: 1.398–8.969, *p*: 0.01) (Table 4). Additionally, in this model, it was identified that the presence of HT increased the risk of aPwa by 2.710-fold (hazard ratio: 2.710, 95% confidence interval: 1.079–6.804, *p*: 0.03)

When Model-2 was analyzed, it was identified that the established binary logistic regression model was significant (Naegele R^2^ = 0.236; *p* < 0.001). According to this model, it was determined that a 1-point increase in the recovered COVID-19 subjects’ chest CT severity score increased the risk of aPwa by 0.896-fold (hazard ratio: 0.896, 95% confidence interval: 0.840–0.955, *p* < 0.001) (Table 5).

## 4. Discussion

This study showed that those individuals who had contracted and recovered from severe COVID-19 had a significantly higher P-wave axis deviation compared to subjects who had never contracted COVID-19 or those who had contracted COVID-19 and subsequently recovered from a mild or moderate case of the disease. The correlation and regression analyses revealed that recovering from severe COVID-19 and the severity score of the recovered COVID-19 subjects’ chest CT were correlated with the presence of P-wave axis deviation and were also predictors for the presence of an abnormal P-wave axis, together with HT (Table 3, Table 4 and Table 5). However, it was also detected that the deviation in the P-wave axis was not related to the number of positive PCR tests obtained for COVID-19 or to the time elapsed after recovery from COVID-19 (Table 3).

**Figure 3:**
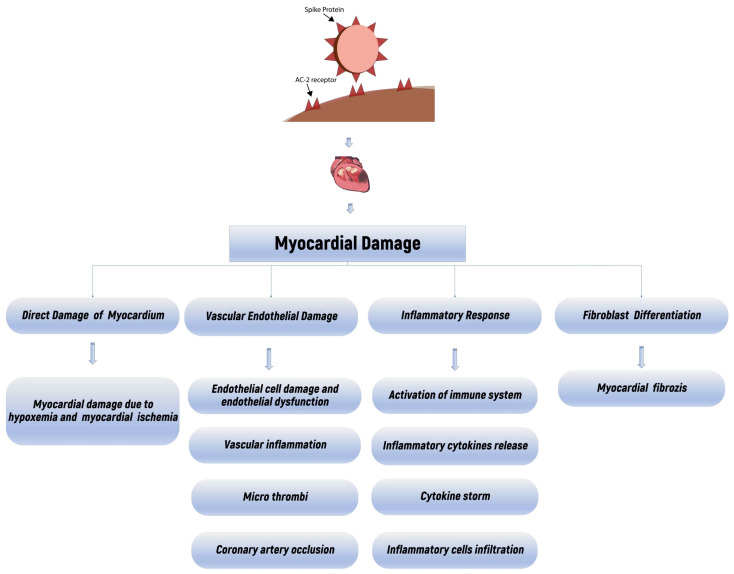
Schematic representation of myocardial damage in COVID-19.

SARS-CoV-2 enters tissues via the spike proteins on the virus surface binding to the angiotensin-converting enzyme 2 (ACE-2) receptor. In humans, the ACE-2 protein is found in many tissues, especially type-2 pneumocytes, myocardium, gastrointestinal tract (GIS), bone marrow, kidney, epithelial cells, central nervous system, and spleen, which explains why SARS-CoV-2 progresses causing multiple organ damage [13]. The possible multiple mechanisms of cardiac damage seen in COVID-19 patients can be summarized as follows: First, a cytokine storm and multiple organ failure resulting from an acute systemic inflammatory response occurs. An imbalance between myocardial oxygen demand and delivery secondary to severe hypoxia resulting from acute respiratory failure follows. Cardiotoxicity that may develop as a result of the treatment agents occurs. This is followed by a tendency to arrhythmia caused by the virus through the ACE-2 signaling system, which causes electrolyte imbalance and embolic or micro-embolic complications that develop as a result of a tendency to thrombosis due to systemic inflammation. Finally, myocarditis possibly develops as a result of the virus entering the cell by binding directly to the ACE-2 receptor causing changes in the ACE-2 signaling system, which is predominantly expressed in the lung and heart tissue (Figure 3) [14,15,16,17,18,19,20,21].

Among the drugs used for the treatment of COVID-19, corticosteroids stand out as the agents used for the longest period (cortisone, dexamethasone, etc.). Corticosteroids can be given in very high doses for a long time, especially to intensive care patients [22]. Corticosteroids are responsible for both mineralocorticoid (MC) and glucocorticoid effects. Mineralocorticoids increase collagen secretion through the activation of fibroblasts; therefore, myocardial diffuse fibrosis may occur in cases of hypercortisolism [23,24].

The P-wave axis illustrates the net direction of atrial depolarization and several conditions that may cause P-wave axis deviation (aPwa). The autonomic nervous system (ANS) dysfunction, pathological left atrial remodeling (such as left atrial dilatation), myocardial ischemia, myocardial fibrosis, and interatrial conduction block due to fibrosis in the area of Bachmann’s bundle could all cause supraventricular arrhythmia. This supraventricular arrhythmia can be detected via P-wave axis deviation using an ECG [8,25,26]. In parallel, as previously known, the existence of aPwa may also predict the risk of atrial fibrillation and all-cause mortality [25,26].

The use of the term “post-COVID syndrome” was defined as a result of patient complaints persisting for about 12 weeks and those symptoms not being explained by another illness after having experienced an acute COVID-19 infection. The common symptoms include palpitations, persistent fatigue, a change in or loss of smell, and chest and muscle pain [27,28,29]. Cardiopulmonary symptoms, such as dyspnea, palpitations, limited physical capacity, dizziness, and presyncope, may persist for weeks to months after acute COVID-19 in a significant proportion of patients (ranging from approximately 10% to 50%) [29,30]. An increasing number of studies are now seeking evidence that these symptoms may underlie subsequent cardiac arrhythmias in patients who were infected with SARS-CoV-2 [31]. Post-COVID-19 symptoms have been reported as occurring more frequently in subjects who have recovered from severe COVID-19. In particular, cardiac symptoms (such as chest pain, dyspnea, orthopnea, palpitations, dizziness, and fatigue) may emerge after acute SARS-CoV-2 infection and persist for months [32]. Recently, some studies have reported complaints of persistent palpitation following COVID-19 [33,34]. The nature and type of cardiac arrhythmia detected with routine electrocardiography, as well as patients’ descriptions of the sensation of palpitations, vary depending on the accuracy and design of the questionnaire used in the study. In most cases, this represents no more than a subjective assessment of the comfort of the patients surveyed. Some recent studies have conducted an online survey and reported that palpitations and tachycardia complaints were detected more in COVID-19 patients than in individuals without a history of COVID-19 [27,35]. These survey results have been interpreted so that postural orthostatic tachycardia syndrome (POTS) was deemed to be responsible for the symptoms expressed by the participants [35]. However, there is a clear need to conduct research on objective electrocardiographic evidence so that these subjective complaints may indicate specific cardiac arrhythmias. From a realistic perspective, most post-COVID-19 studies have attributed their conclusions to surveys based on participants’ subjective complaints and have not been designed to detect specific arrhythmias, such as ventricular and/or supraventricular arrhythmias [36]. On the other hand, a retrospective study conducted to shed light on this issue revealed that COVID-19 patients treated without hospitalization had a 1.7-fold higher incidence of AF at 6 months after previous SARS-CoV-2 infection compared to controls without a history of COVID-19 infection [37]. However, this study did not allow a distinction to be made as to what proportion of patients had atrial fibrillation that could not be detected before hospitalization, or which systemic (e.g., diabetes mellitus or rheumatoid arthritis) or cardiac conditions (such as the presence of structural heart disease detectable only by transthoracic echocardiography) were present that could cause atrial fibrillation, and therefore, which patients had an active COVID-19 infection that caused atrial fibrillation. Additionally, this methodology makes it difficult to distinguish between cases in which acute atrial fibrillation triggered by acute COVID-19 turns into permanent atrial fibrillation and patients with atrial fibrillation where the true cause is post-COVID-19 syndrome.

Could the neurological, cardiac, and systemic effects of COVID-19 itself and the drugs used for the treatment (especially corticosteroids) cause a permanent predisposition for supraventricular arrhythmia via possible left atrial damage and/or ANS dysfunction? The present study showed that the probability of detecting aPwa on superficial ECG was higher in the subgroup that recovered from severe COVID-19 in comparison to the control group and the other subgroups. Furthermore, this study revealed that recovery from severe COVID-19 and the chest CT severity score of the recovered COVID-19 subjects were independent predictors of aPwa alongside hypertension (Table 2, Table 3, Table 4 and Table 5). Previous studies had shown that hypertension and advanced age both caused an abnormal P-wave axis [8,9,26,38]. Although the current study also indicated that HT causes aPwa, there was no significant difference in reported HT between the subgroups, and in regression analyses, age does not reach statistically significant values. In addition to these results, the significant results of recovery from severe COVID-19 and the severity score of recovered COVID-19 subjects’ chest CT suggest that exposure to severe SARS-CoV-2 infection may be one of the main causes of aPwa. Additionally, correlation analysis showed that the number of positive PCR tests for COVID-19 and the time elapsed after infection with COVID-19 were not associated with the presence of aPwa. Therefore, the results suggest that recovery from severe COVID-19 may cause a permanent abnormal P-wave axis (Table 3). These findings can be attributed to the medical and invasive interventions in the treatment algorithm of severe COVID-19 patients, as well as to the nature of the SARS-CoV-2 virus, which is known to damage the myocardium and ANS during acute illness [13,14,15,16,17,18,19,20,21,22,23,24,39].

The data show that subjects who recovered from severe COVID-19 are more likely to permanently experience aPwa and therefore have a higher risk of developing supraventricular arrhythmia than those who have either never had COVID-19 or did not experience severe COVID-19. This study demonstrated that an abnormal P-wave axis was independently associated with recovering from severe COVID-19, suggesting the need to perform a diagnostic electrocardiographic examination among subjects who have recovered from severe COVID-19 for the early detection of abnormal P-wave axis and to prevent more severe supraventricular arrhythmias.

## 5. Conclusions

The fact that patients who had been diagnosed and had recovered from COVID-19 were admitted to the hospital with complaints of palpitations that had not dissipated several months post-infection initiated the hypothesis that the process of COVID-19 infection may cause permanent damage to the myocardium itself, to the intrinsic conduction pathways, or the ANS. Any damage that may occur in the ANS, myocardial tissue, or the intrinsic conduction pathways should be noted as a possible cause of arrhythmia. For this reason, the presence of aPwa detectable on ECG, which is a cost-effective tool, reveals that recovered COVID-19 cases presenting with palpitations should be evaluated with a detailed cardiac examination including advanced examinations such as 24-hour ECG Holter monitoring. The P-wave axis deviation, which can easily be detected on the ECG by physicians in all specialties, can provide an important guiding marker for further examination to detect the presence of supraventricular arrhythmia. In recently published articles on the term “post-COVID-19”, we observed that palpitation complaints have been attributed to panic disorder, anxiety, or depression, and a parallel treatment algorithm has been added to the daily routine [40,41,42,43,44]. The results in this study suggest that severe COVID-19 may lead to permanent aPwa and therefore a permanent predisposition to increased risk of supraventricular arrhythmias in patients who have recovered from severe COVID-19. Therefore, this study provides concrete evidence that continued patient complaints, months after recovery from COVID-19, may indicate symptoms of cardiac origin. In this context, adding ECG to the routine physical examination algorithm and performing P-wave axis evaluation in subjects who have recovered from severe COVID-19 and who apply to outpatient clinics may help in identifying subjects at high risk of supraventricular arrhythmias.

## Figures and Tables

**Table 1:** Semi-quantitative chest computed tomography (CT) severity score and chest CT severity index.

Individual Lobar Scores Based on Percentage of Involvement		COVID-19 Chest CT Severity Index Based on the Involvements of the Five Lobes	
Lobar Involvement	Score	Total Score	Severity (Category)
0–5%	1	<8	Mild
5–25%	2	8–15	Moderate
26–49%	3	16–25	Severe
50–75%	4		
>75%	5		

**Table 2:** Comparison of demographic variables and aPwa between recovered COVID-19 subgroups and control group.

	The Controls (Noun: 251)	Subgroup I (Recovered Mild COVID-19 Group, Noun: 142)	Subgroup II (Recovered Moderate COVID-19 Group, Noun: 57)	Subgroup III (Recovered Severe COVID-19 Group, Noun: 53)	*p*
Age (Years)	45.0 (26.0–55.0)	44.0 (32.75–51.0)	46.0 (39.50–55.50)	50.0 (41.0–55.0)	^a^ 1.0,^b^ 0.29, ^**c**^ **<0.001**, ^**d**^ **0.02**, ^**e**^ **<0.001**, ^f^ 0.68
Gender (Female) (%)	52.6	50.0	47.4	41.5	^a^ 0.62, ^b^ 0.57, ^c^ 0.19, ^d^ 0.86, ^e^ 0.29, ^f^ 0.67
HT (%)	23.1	23.2	31.6	35.8	^a^ 0.97, ^b^ 0.24, ^c^ 0.78, ^d^ 0.30, ^e^ 0.11, ^f^ 0.78
Smoking (%)	30.3	34.5	38.6	37.7	^a^ 0.39, ^b^ 0.29, ^c^ 0.37, ^d^ 0.70, ^e^ 0.80, ^f^ 1.00
aPwa (%)	(4.0%)	(5.6%)	(7.0%)	(22.6%)	^a^ 0.61, ^b^ 0.30, ^**c**^ **<0.001**, ^d^ 0.74, ^**e**^ **0.001**, ^**f**^ **0.04**

a: *p* value between control group and Subgroup 1.; b: *p* value between control group and Subroup 2; c: *p* value between control group and Subgroup 3; d: *p* value between Subgroup 1 and Subgroup 2; e: *p* value between Subgroup 1 and Subgroup 3; f: *p* value between Subgroup 2 and Subgroup 3.

**Table 3:** Spearman’s Rho correlation analysis between abnormal P-wave axis, recovered COVID-19 subgroups, post-COVID-19 recovery duration, number of positive PCR tests for COVID-19, and subject’s chest CT severity score.

	Abnormal P-Wave Axis	
	R	*p*
Recovered COVID-19 subgroups	−0.194	0.002
Post COVID-19 recovery duration (week)	0.047	0.461
Number of positive PCR tests for COVID-19	−0.110	0.081
Recovered COVID-19 subject’s chest CT severity score	−0.245	<0.001

**Table 4:** Binary logistic regression for variables (dependent variable: abnormal P-wave axis).

	*β*	S.E.	Hazard Ratio	95% Confidence Interval		*p*
HT	0.997	0.470	2.710	1.079	6.804	0.03
Smoking	0.561	0.469	1.753	0.699	4.400	0.23
Age	−0.028	0.023	0.973	0.929	1.018	0.23
(Recovered Severe COVID-19 Subgroup)	1.265	0.474	3.542	1.398	8.969	0.01
Gender	0.455	0.482	1.576	0.613	4.054	0.34
Post COVID-19 recovery duration (week)	0.007	0.008	1.007	0.992	1.022	0.38
Number of positive PCR tests for COVID-19	−0.418	0.384	0.658	0.310	1.398	0.27
Constant	−1.422	2.144	0.241			0.50

*p* < 0.001; Nagelke R^2^ = 0.195.

**Table 5:** Model-2. Binary logistic regression for variables (dependent variable: abnormal P-wave axis).

	*β*	S.E.	Hazard Ratio	95% Confidence Interval		*p*
HT	1.003	0.479	2.725	1.065	6.972	0.03
Smoking	0.600	0.483	1.823	0.707	4.701	0.21
Age	−0.023	0.024	0.977	0.932	1.023	0.32
Recovered COVID-19 subject’s chest CT severity score	−0.110	0.033	0.896	0.840	0.955	<0.001
Gender	0.378	0.491	1.460	0.558	3.819	0.44
Post COVID-19 recovery duration (week)	0.005	0.008	1.005	0.991	1.020	0.48
Number of positive PCR tests for COVID-19	−0.505	0.389	0.603	0.282	1.293	0.20
Constant	1.977	1.919	7.218			0.30

*p* < 0.001; Nagelke R^2^ = 0.236.

## Data Availability

The datasets used and/or analyzed during the current study are available from the corresponding author on reasonable request.
